# Comparative optimism in models involving both classical clinical and gene expression information

**DOI:** 10.1186/1471-2105-9-434

**Published:** 2008-10-15

**Authors:** Caroline Truntzer, Delphine Maucort-Boulch, Pascal Roy

**Affiliations:** 1 Hospices Civils de Lyon, Service de Biostatistique, Lyon, France; 2 Université Claude Bernard Lyon 1, Université de Lyon, Villeurbanne, France; 3 Laboratoire Biostatistique Santé, UMR CNRS 5558, Pierre-Bénite, France; 4 Clinical and Innovation Proteomic Platform, CHU Dijon, France

## Abstract

**Background:**

In cancer research, most clinical variables have already been investigated and are now well established. The use of transcriptomic variables has raised two problems: restricting their number and validating their significance. Thus, their contribution to prognosis is currently thought to be overestimated. The aim of this study was to determine to what extent optimism concerning current transcriptomic models may lead to overestimation of the contribution of transcriptomic variables to survival prognosis.

**Results:**

To achieve this goal, Cox proportional hazards models that adjust for clinical and transcriptomic variables were built. As the relevance of the clinical variables had already been established, they were not submitted to selection. As for genes, they were selected using both univariate and multivariate methods. Optimism and the contribution of clinical and transcriptomic variables to prognosis were compared through simulations and by using the Kent and O'Quigley *ρ*^2 ^measure of dependence. We showed that the optimism relative to clinical variables was low because these are no longer submitted to selection of relevant variables. In contrast, for genes, the selection process introduced high optimism, which increased when the proportion of genes of interest decreased. However, this optimism can be decreased by increasing the number of samples.

**Conclusion:**

Two phenomena have to be taken into account by comparing the predictive power and optimism of clinical variables and those of genes: overestimation for genes due to the selection process and underestimation for clinical variables due to the omission of relevant genes. In comparison with genes, the predictive value of validated clinical variables is not overestimated, which should be kept in mind in future studies involving both clinical and transcriptomic variables.

## Background

In the field of cancer research, classical clinical variables have long been used as prognostic markers. Indeed, many strong clinical determinants that explain most of the prognosis have already been identified. Nevertheless, certain characteristics of cancer are still poorly understood, and these need to be elucidated to improve treatment. Thus, cancer research is making use of new technologies, especially microarrays, and survival analysis methods have been extended to take into account the potential information from microarray analysis with the hope that this transcriptomic information will supersede clinical data. For example, Shipp *et al. *[1] showed that a 13-genes-signature-based outcome predictor provided additional information not reflected in the clinical prognostic model based on the International Prognostic Index. Today, cancer clinicians would like to combine genes and classical clinical variables in the same models to improve assessment of cancer prognosis. In the recent years, some authors addressed this question in two ways. The first way is to involve classical clinical and transcriptomic variables in the same models [2-4]. However, not all authors took into account the particularity of each type of variable. The second way is to consider the additional predictive value of genes. In this context, Tibshirani and Efron [5] warned in 2002 against too premature conclusions regarding the predictive power of transcriptomic variables. Using the breast study from Van't Veer [6], those authors showed that the effects of genes were overestimated with regard to the classical clinical variables like tumor grade or size. They proposed a "pre-validation" strategy to correct the artificial importance given to genes.

In the very recent literature, few authors joined those two ways in unique models. Thus, Binder and Schumacher [7] proposed an offset-based boosting approach in the context of survival data. This approach allows also answering the question whether prediction is improved or not by adding transcriptomic variables to classical clinical variables in the same model. Shortly later, Boulesteix et al. [8] proposed a more general approach than that of Binder and Schumacher, in the sense that it was not limited to survival data. This approach is based on PLS, random forest, and the pre-validation strategy suggested by Tibshirani and Efron.

The interesting thing in the two above approaches is that they allow simultaneously to construct a classifier combining both types of variables and to determine whether microarray data present additional predictive value.

In agreement with this consideration, we think it is of major importance when designing statistical models to keep in mind that the characteristics of transcriptomic variables are completely different from those of clinical variables. Specific clinical variables have already been validated in many large studies. Thus, most of these clinical variables are no longer included in the selection process (e.g. the estrogen receptor status in breast cancer or the international prognostic index in lymphoma). In contrast, the selection step is still needed for genes, and various issues are still a matter of debate. First, fewer studies have been conducted on transcriptomic variables than on clinical variables; thus, there are fewer datasets available to repeat the analyses and validate the relationships. In most cases, genes selected in a single study are assumed to have a general prognostic value. This selection is presented as a benchmark for the disease without external validation studies on new datasets. Second, whenever available, these datasets are rather small compared to the number of genes under study. Considering the high number of variables and the relatively low number of observations, microarray data can easily lead to a high number of false-positive variables. By chance alone, many genes may be found significantly associated with the outcome even though most of them may not actually be linked to prognosis.

Some publications have clarified certain additional issues related to the selection process in microarray analysis. EinDor et. al showed that the final gene signature depends highly on the subset of patients used for gene selection [9]. Later, the same team pointed out that the reproducibility of a signature depends on the number of samples used for the analysis [10]. Other teams were interested in the False Discovery Rate (FDR); that is, the expected proportion of false positives among the genes declared as significant. When looking for differential genes, Pawitan et. al showed that the FDR is mostly influenced by the proportion of truly differentially expressed genes and by the sample size [11].

The same problems are met in survival studies where the construction of transcriptomic models raises simultaneously the problem of restricting the number of genes to include and that of validating the selected genes. When a model is too complex – i.e., the number of free parameters to estimate is too high given the information contained in the data – the strength of that model will be exaggerated due to overfitting. Some conclusions of the analysis may be due to noise or to some spurious associations between the covariates and the outcome. In this case, the model has high adequacy and predictive accuracy for the dataset on which it was built but is not able to accurately predict the outcome with new datasets. Thus, the ability of the model to predict outcome with new datasets is overestimated: this is called optimism. The main objective of this article is to quantify to what extent the optimism of transcriptomic models induced by the selection process may lead to overestimation of the contribution of transcriptomic variables to prognosis, especially in comparison with clinical variables when they are included in a single model. To be able to control the contribution of the two types of variables, the following study was conducted on simulated datasets.

## Methods

To compare optimism relative to clinical and transcriptomic models within the context of survival, the study was based on simulated datasets that included both clinical and transcriptomic variables. We wanted to simulate the "real situation" faced by clinicians and statisticians: classical clinical variables are already validated, whereas transcriptomic variables are still in the selection and validation process. Regarding clinical variables, only validated ones are considered to build combined classifiers, while regarding genes, many of the considered ones are still superfluous noise genes.

To analyze one dataset, the following procedure was employed: (1) The variables of interest were first identified; (2) Once clinical variables or genes had been chosen, Cox proportional hazards models including both types of variables were constructed; (3) To measure the predictive information contained in each survival model, the *ρ*^2 ^measure of dependence from Kent and O'Quigley was used so that optimism for both types of variable could be compared [12,13].

R and S-Plus codes used in our analyses are available at .

### Simulation of the datasets

A classical way to link variables to censored survival data is to use the Cox proportional hazards model. Let us define *X *an (*n*, *m*) matrix of *m *variables for *n *individuals. For each of the *n *patients, the follow-up times were noted *t*_1_,..., *t*_*n *_as were the event-indicators *d*_1_,..., *d*_*n *_with *d*_*i *_= 1 if the event occurred and *d*_*i *_= 0 if it did not occur. At time t, the Cox proportional model is given by:

(1) *λ*(*t*|X) = *λ*_0_(*t*)*exp*(*β*'**X**)

where *λ*_0_(*t*) is a baseline hazard function, *β *= {*β*_1_,..., *β*_*m*_} is the vector of parameters and *X*_1_,..., *X*_*m *_are the vectors of length *n *describing each of the *m *variables for the *n *patients.

Through this model, we simulated a virtual population of size *n *in which the *m *variables consisted of both clinical and transcriptomic variables. The simulation process was inspired from the simulation study from Gui and Li [14], which R code is publicly available.

More precisely, each patient was described by two clinical variables, *p *genes and survival information. The aim was to estimate in a single model the relationship between the two types of variables and survival times.

Clinical variables were simulated using binomial distributions with probabilities 0.5 and 0.4, respectively, as parameters for success (e.g. the positive vs. negative estrogen status). Normal distributions *N*(0, 1) were assumed for the transcriptomic variables. A Weibull distribution with shape parameter 5 and scale parameter 2 was used for the baseline function. For censoring times, a uniform *U*(0, 8) was used, leading to about 40% censoring. The underlying model was:

(2) $$\lambda (t|X_{C},X_{T})=\lambda_{0}(t)\exp({\beta^{\prime }}_{C}X_{C}+{\beta^{\prime }}_{T}X_{T})$$

where *X*_*C *_and *X*_*T *_were respectively the matrix describing the clinical and the transcriptomic variables.

As there were two clinical variables, *X*_*C *_was an (*n*, 2) matrix. As for genes, *p *were under study, leading to an (*n*, *p*) matrix *X*_*T*_. Only *p*_1 _of the *p *genes were considered as related to survival; the *p*_0 _remaining genes were under the null hypothesis *H*_0 _of no association with survival. Note that *p *= *p*_1 _+ *p*_0 _and *m *= *p *+ 2. The relevance of most of the clinical variables had already been established through several studies. The two clinical variables were then considered significant, and coefficients for both of these variables were set at 0.8: *β*_*C*, *i *_= 0.8, *i *= 1, 2. Coefficients for transcriptomic variables related to survival were set at 0.2: *β*_*T*, *i *_= 0.2, *i *= 1,..., *p*_1 _and the remaining *p*_0 _were set at 0: *β*_*T*, *i *_= 0, *i *= *p*_1 _+ 1,..., *p*.

For a fixed set of parameters *p *and *p*_1_, *r *= 60 training sets of *n *patients were simulated according to the design described above. For each of these training sets, 50 corresponding test sets were drawn following the same design. This overall process was performed varying *n*, *p *and *p*_1 _sequentially. The number of patients *n *was considered in {50, 100, 200, 400}, *p *was considered in {500, 1000, 2000, 4000} and *p*_1 _was considered in {5, 10, 20}.

A single Cox proportional hazards model involving both clinical and transcriptomic variables could then be estimated for each of these simulated datasets, as described below.

### Variable selection and model construction

#### Cox proportional hazards model

In the traditional Cox model, the vector of parameters is such that it maximizes the following Cox partial likelihood (PL):

(3) $$PL(\beta )=\prod_{k\in D}\frac{exp(\beta^{\prime }x_{k})}{\sum_{j\in R_{k}}exp(\beta^{\prime }x_{j})}$$

where *D *is the set of indices of the events and *R*_*k *_is the set of indices of the individuals at risk at time *t*_*k*_.

In our case, there were *p *+ 2 parameters to estimate. This leads to a huge number of variables in comparison with the number of individuals; the high dimensional space of the transcriptomic predictors thus precludes the use of the standard maximum Cox partial likelihood method to estimate the parameters. Several methods have been proposed in the literature to deal with this high dimension issue in survival models involving genes.

#### Adaptation to high-dimensional data

The first solution aims at selecting a lower subset of genes according to the relevance of each gene taken separately. This approach takes each feature in an univariate way and also does not take into account the interactions between genes. We used the log-rank statistic to order genes; the number of genes to be involved in the model was chosen a priori. We retained the 20 genes with the highest statistical values, so that the number of selected genes was in the same order of magnitude as in the approach which follows. The second solution is a multivariate selection method that simultaneously selects genes and estimates their effect on survival. One way to do this is to maximize the partial likelihood under constraints using *L*1 or *L*2 penalization. Contrary to the *L*2 penalization [15,16], which uses all genes in the prediction, only some genes are used in the prediction with the *L*1 penalization [17]. The threshold gradient descent (TGD) method proposed by Friedman and Popescu [18] allows a compromise between the *L*1 and *L*2 penalizations. Through the choice of a defined threshold, it approximates the *L*2 (low threshold) and *L*1 (high threshold) penalized estimations. Gui and Li [19] extended the TGD to the survival model and demonstrated the ability of their approach to select relevant genes and to provide good predictive performance. We therefore used this model to select the genes to include in our models.

Briefly, the TGD method is based on the gradient method, which is classically employed to determine the minimum of a loss function. With this method, the parameters vector is derived in a sequential manner following the direction of the negative gradient of the loss function, here the partial log-likelihood noted *l*(*β*_*T*_) and defined as *l*(*β*_*T*_) = -*logPL*(*β*_*T*_). The negative gradient is defined as: **g**(*ν*) = -∂*l*/∂*β*_*T *_Starting with ${\hat{\beta }}_{T}$ = 0, the vector of estimated parameters ${\hat{\beta }}_{T}$ is then updated at each iteration:

$${\hat{\beta }}_{T}(\nu +\Delta \nu )={\hat{\beta }}_{T}(\nu )+\Delta \nu .h(\nu )$$

The parameter *ν*, which begins at zero, controls the number of iterations. Δ(*ν*) controls the incremental movement along the gradient. *h*(*ν*) is defined as: $h(\nu )={\left\{f_{j}(\nu )\cdot g_{j}(\nu )\right\}}_{1}^{p}$, with *f*_*j*_(*ν*) = *I *[|*g*_*j*_(*ν*)| ≥ *τ*.max_1≤*k*≤*p*_|*g*_*k*_(*ν*)|], *I *[.] being the indicator function, and *τ *∈ [0, 1] a user-defined constant. Through **f**(*ν*), only coefficients for which the gradient exceeds the threshold determined by *τ *are updated at each step. The final model is given by the value of *ν *which minimizes the cross-validated partial log-likelihood (CVPL).

The final vector of parameters ${\hat{\beta }}_{T}$ has only one piece of non-null coefficients that corresponds to genes that are relevant to predict survival.

The number *p*_2 _of non-null coefficients depends on the choice of *τ*. Note that the set of the *p*_2 _genes selected by the TGD may differ from the initial *p*_1 _set of simulated genes we considered linked to survival. With *τ *= 0, all genes are kept in the final model. Thus, all the predictive variables are selected but, in return, all the noisy variables are also selected. In contrast, with *τ *= 1, only one gene is kept at each iteration. This time, only a restricted number of genes is selected. Among these selected genes, the majority is actually predictive but, in return, some important variables are missed. We have chosen *τ *= 0.8, which allows finding a compromise between the two extreme situations obtained with *τ *= 0 and *τ *= 1. This choice leads to a limited number of selected genes: between 20 and 40 genes with our simulated datasets, which is a reasonable number of selected genes regarding the number of genes simulated under H0.

Although the TGD method combines selection of genes and estimation of their effect on survival, we used it only for selection purposes; that is, to select genes irrespective of their estimated coefficients.

Thus, we used two approaches to select genes: a univariate approach using the log-rank, and a multivariate approach based on the TGD. As for clinical variables, they were considered as validated and, thus, directly included in the final model.

The optimism arising respectively from the clinical and the transcriptomic variables was then estimated and compared as follows.

### Comparison of the contribution of the variables to the prognosis

#### *ρ*^2 ^as a measure of explained variability

Different criteria allow selection and comparison of models based on their capacity to predict the outcome of individuals who did not participate in the model building. Among them, explained variability reflects the robustness of the model, and its efficiency in predicting outcomes on new datasets. It measures the information given by some variables involved in a specific model.

In linear regression, the coefficient of determination *R*^2^quantifies the proportion of variability in a data set that is accounted for by a statistical model. Kent and O'Quigley proposed rewriting the *R*^2 ^based on the Kullback-Leibler Information [20], which quantifies the information gain brought by variables involved for example in a Cox Proportional Hazards model. This measure is defined as:

$$\rho^{2}=1-exp\left[-2(I(\hat{\beta })-I(0))\right]$$

where 2(*I*($\hat{\beta }$) - *I*(0)) quantifies the difference between information from the model with $\hat{\beta }$ estimated vector and the null model with no covariables. *ρ*^2 ^is comprised between 0 and 1, with *ρ*^2 ^= 0 for the null model and *ρ*^2 ^→ 1 when all parameters tend to infinity.

#### Application

We used this measure to quantify the optimism arising from the clinical and transcriptomic variables. For this, three *ρ*^2 ^with different meanings were computed: $\rho_{Pop}^{2}$, $\rho_{Tr}^{2}$ and $\rho_{Te}^{2}$. These values were computed respectively for each type of variable in the model involving both of them.

$\rho_{Pop}^{2}$ reflects the information accounted for by the variables in the virtual global population. It is computed using the *β*_*T *_vector of coefficients defined earlier in the simulation process. Only the *p*_1 _non null parameters contribute to its computation. $\rho_{Pop}^{2}$ therefore only depends on *p*_1 _and has the same value whatever *n *and *p*. In contrast, $\rho_{Tr}^{2}$ and $\rho_{Te}^{2}$ take into account the selection and the estimation processes. More precisely, $\rho_{Tr}^{2}$ reflects the information accounted for by the variables selected on one specific training set sampled from the global population. It is computed using the *p*_2 _coefficients of the model estimated on the training sets. $\rho_{Te}^{2}$ reflects the information accounted for on test sets by variables selected on the training set. It is computed using coefficients estimated on the test sets. We used ${\bar{\rho }}_{Te}^{2}$, which is the mean of the $\rho_{Te}^{2}$ computed on the 50 test sets associated with each training set.

As for $\rho_{Te}^{2}$, coefficients of genes selected on the training set were estimated on the test set. In fact, the computation of *ρ*^2 ^only requires the knowledge of the coefficients of the Cox model and the distribution of the corresponding predictive variables. By re-estimating the coefficients of the Cox model on the test set to compute $\rho_{Te}^{2}$, we are able to evaluate the predictive information actually given in the test set by the genes selected on the training set. A lack of re-estimation of the coefficients would have assumed that the predictive power of the genes selected on the training set-given by the coefficients of the Cox model estimated on the training set- is equal on the test set. The selection process introduces much optimism. The latter must be taken into account by re-estimating the coefficients on the test set. By doing so, the predictive information of the selected genes will be closer to their effective predictive information in the test set.

These measures were estimated for both the clinical and the transcriptomic variables.

#### Optimism quantification

Optimism was quantified by computing relative differences between the various *ρ*^2^, as described in equations 4 to 6. By comparing *ρ*^2 ^values estimated in the training and the test sets, Δ_*TrTe *_(Equation 4) shows the error made by considering that the signature given by one dataset is the real signature and delivers the same information on other datasets. In other words, it gives the difference between the predictive information anticipated on one dataset and the effective information on another.

Δ_*TrPop *_(Equation 5) and Δ_*TePop *_(Equation 6) compared respectively $\rho_{Tr}^{2}$ and ${\bar{\rho }}_{Te}^{2}$ with $\rho_{Pop}^{2}$. Both measures quantify the relative difference between effective predictive information in one dataset and the detected one. Comparing $\rho_{Pop}^{2}$ to $\rho_{Tr}^{2}$ quantifies how the predictive information thought to be contained in one training set moved away from information contained in the whole population it is sampled from. It allows the validation process to be evaluated. Comparing $\rho_{Pop}^{2}$ to ${\bar{\rho }}_{Te}^{2}$, allows the selection process to be evaluated.

The three following measures were computed for each *i *= 1,..., 60 training sets simulated with each combination of the parameters *p*, *p*_1 _and *n*.

(4) $$\Delta_{TrTe,i}=\rho_{Tr,i}^{2}-\frac{\sum_{j=1}^{50}\rho_{Te,ij}^{2}}{50}=\rho_{Tr,i}^{2}-{\bar{\rho }}_{Te,i}^{2}$$

(5) $$\Delta_{TrPop,i}=\frac{\rho_{Tr,i}^{2}-\rho_{Pop,i}^{2}}{\rho_{Pop,i}^{2}}$$

(6) $$\Delta_{TePop,i}=\frac{1}{\rho_{Pop,i}^{2}}\left[\frac{\sum_{j=1}^{50}\rho_{Te,ij}^{2}}{50}-\rho_{Pop,i}^{2}\right]=\frac{{\bar{\rho }}_{Te,i}^{2}-\rho_{Pop,i}^{2}}{\rho_{Pop,i}^{2}}$$

## Results

Results are shown through boxplots. We found this way of representation well-suited to show the distribution of the various differences obtained over each set of 60 training datasets. Each point contributing to the boxplot corresponds to one measure of Δ, one Δ being computed for each of the 60 training sets.

### Number of patients

Results are shown in an example with *p *= 1000 genes. For the clarity of the figure, only results obtained with *p*_1 _= 10 are shown. They remain the same with *p*_1 _= 5 or *p*_1 _= 20.

**Figure 1:**
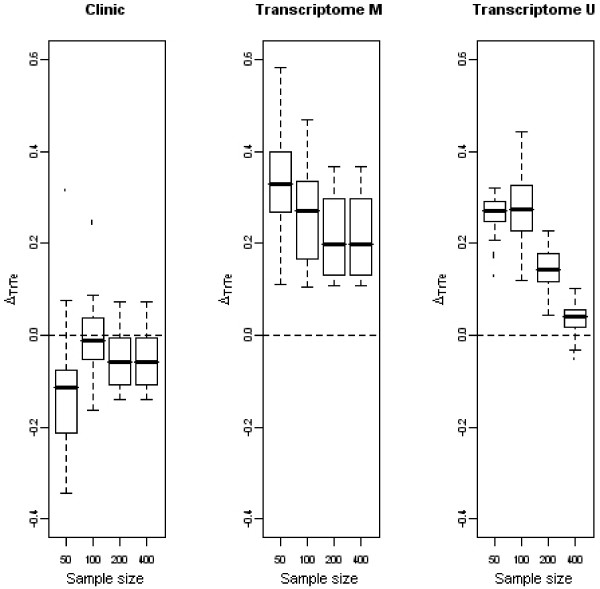
**Evolution of Δ_*TrTe *_with the sample size**. Boxplots representative of the evolution of Δ_*TrTe *_with the sample size for the clinical variables (first panel), and the transcriptomic variables selected through the multivariate (second panel), and the univariate (third panel) way. *p *= 1000 genes.

Figure 1 shows the results obtained for Δ_*TrTe *_(differences between $\rho_{Tr}^{2}$ and ${\bar{\rho }}_{Te}^{2}$) for the clinical model and for the two transcriptomic models obtained with the multivariate (transcriptome M) or univariate selection (transcriptome U).

Regarding clinical variables, Δ_*TrTe *_varied around zero and overall did not depend on the sample size. Regarding genes, the difference decreased with increasing sample size; the difference never reached zero in the multivariate case. The decreasing effect was even stronger with the univariate selection method. This can be explained by the fact that the number of selected genes depended on the test set for the multivariate method whereas it is fixed a priori in the univariate method; in the former, the number of selected genes also varied and we observed that this number increased with the number of patients. The TGD selected genes that were not truly related to survival (False Positives) contributed to the computation of $\rho_{Tr}^{2}$ although they were noise. As a consequence Δ_*TrTe *_had higher values for the transcriptomic model in multivariate selection than in univariate selection, even for *n *= 400 patients. These results show that transcriptomic and clinical variables have different behaviors. The predictive power of genes selected on one dataset is overestimated with regard to the predictive power they would have with other datasets. On the contrary, the predictive power of clinical variables is the same with both training and test sets. As a result, the two types of variables cannot be interpreted the same way.

**Figure 2:**
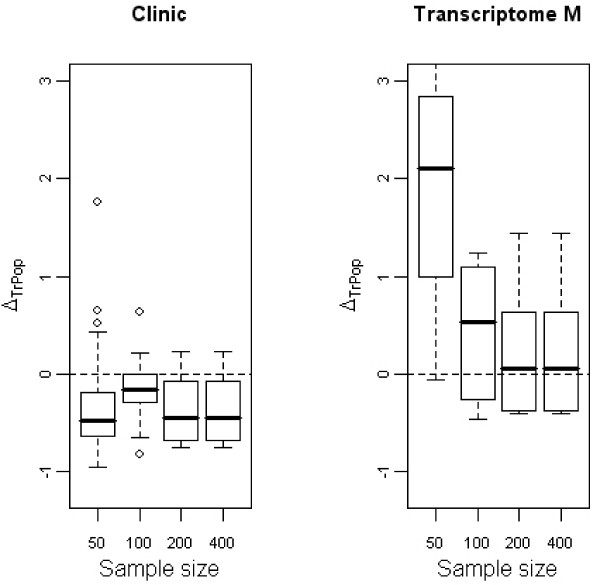
**Evolution of Δ_*TrPop *_with the sample size**. Boxplots representative of the evolution of Δ_*TrPop *_with the sample size for the clinical variables (first panel) and the transcriptomic variables selected through the multivariate way (second panel). *p *= 1000 genes.

Figure 2 shows the differences between $\rho_{Tr}^{2}$ and $\rho_{Pop}^{2}$ with transcriptomic and clinical models. Only results for the transcriptomic variables from multivariate selection are shown.

Regarding clinical variables, their predictive power was clearly underestimated. This observation was amplified with high values of *p*_1 _(results not shown). This can be explained by the bias due to missing covariates when a model is badly specified. This phenomenon is an illustration in the high-dimensional setting of a known bias adjustment phenomenon demonstrated by Chastang in 1988 [21] in the classical context of *n >> p*. Through formulae and simulations studies, the author showed that when explanatory variables are omitted in a non-linear model-multivariate exponential Cox and Weibull survival models- the effect of non-missing covariates is underestimated. This is typically what happens when selecting genes on the training set and when some relevant genes are not detected by the method. Because the TGD method selects nearly the same number of genes whatever *p*_1_, the higher *p*_1_, the greater the number of genes missed at selection. Note that, on the contrary, including non-relevant genes in the model does not bias the estimation of the other covariates.

**Figure 3:**
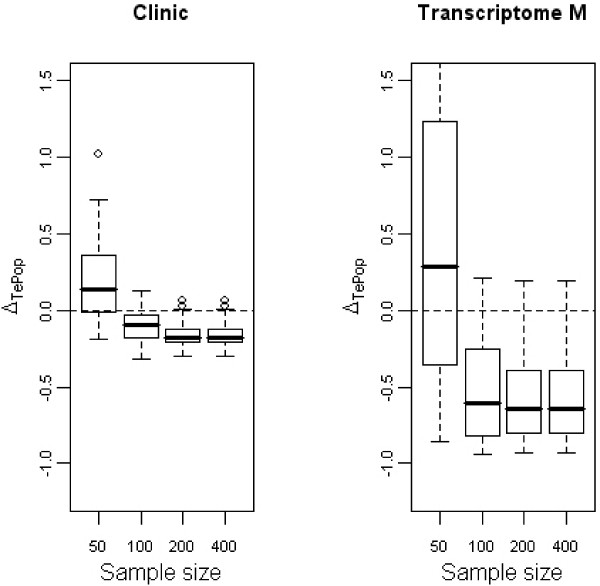
**Evolution of Δ_*TePop *_with the sample size**. Boxplots representative of the evolution of Δ_*TePop *_with the sample size for the clinical variables (first panel) and the transcriptomic variables selected through the multivariate way (second panel). *p *= 1000 genes.

Regarding genes, Δ_*TrPop *_tended to zero when the number of patients increased; the higher the number of patients, the nearer the adjusted $\rho_{Tr}^{2}$ was to the expected $\rho_{Pop}^{2}$. When the sample size is too small, the highly predictive information assumed to be given by the selected genes is far from true information. We were also interested in differences between adjusted ${\bar{\rho }}_{Te}^{2}$ and $\rho_{Pop}^{2}$ (figure 3). Regarding the clinical variables, the results were the same as for the previous differences.

Regarding genes, by using the ones selected with the training set in the test set, the differences Δ_*TePop *_were mainly negative when the sample size exceeded 50 patients: this means that the selected genes were not able to report the true information contained in the dataset. In other words, the genes selected on the previous dataset had no predictive power on other datasets because they were not relevant.

**Figure 4:**
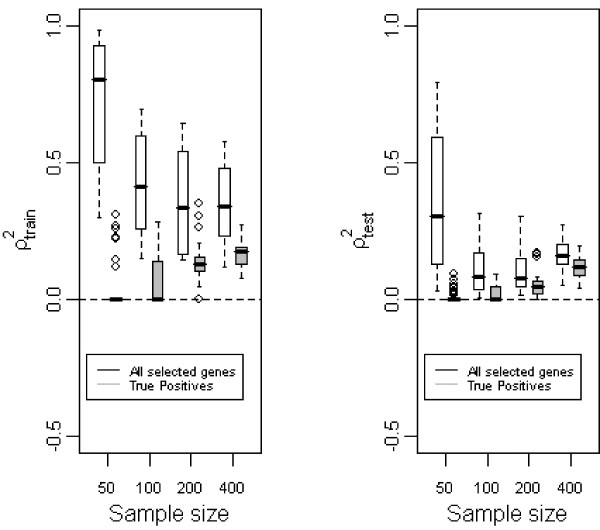
**Role of true positives**. Boxplots representative of the evolution of $\rho_{Tr}^{2}$ (first panel) or ${\bar{\rho }}_{Te}^{2}$ (second panel) with the sample size given that all selected genes or only true positives are taken into account. *p *= 1000 genes.

When selecting the *p*_2 _genes, these can be either really related to survival (genes among the *p*_1 _ones under the alternative hypothesis *H*_1_) or not (genes among the *p*_0 _ones under the null hypothesis *H*_0_). The former are true positives (TP), and the latter false positives (FP). To study the influence of the TP on optimism, we compared the evolution of *ρ*^2 ^due to the TP on the one hand, and to all selected genes on the other hand. This was done with the training and the test sets in the case of multivariate selection of genes. The left panel of figure 4 shows that increasing *n*, $\rho_{Tr}^{2}$ remained of the same order for all selected genes whereas the *ρ*^2 ^due to the TP increased. In contrast, the right panel of figure 4 shows that ${\bar{\rho }}_{Te}^{2}$ for all selected genes or for TP only evolved in the same way. In cases with 50 or 100 patients, there were no TP; $\rho_{Tr}^{2}$ was also only due to noise; this cannot be seen when using only one dataset for a study and may lead to incorrect interpretation of noise as information. The high values observed for all the genes for the 50 patients are due to the fact that there were too few patients to get good estimations.

### Total number of genes

**Figure 5:**
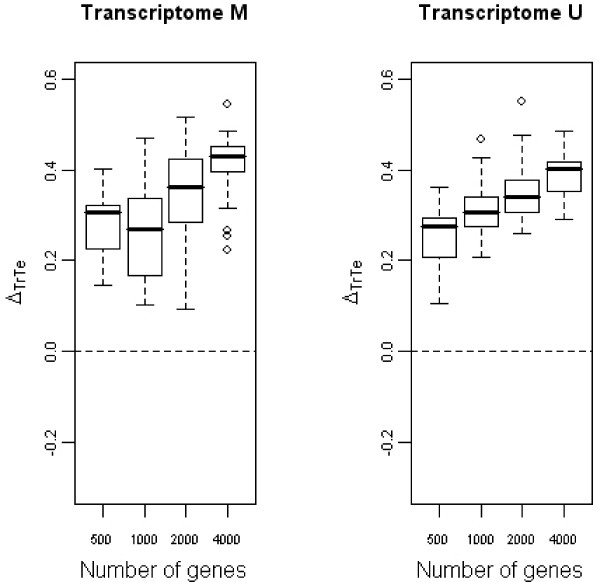
**Evolution of Δ_*TrTe *_with the number of genes under study**. Boxplots representative of the evolution of Δ_*TrTe *_with the number of genes under study for the transcriptomic variables selected through the multivariate (first panel) or the univariate way (second panel). *n *= 100 patients.

The need for a lot of individuals is valid whatever the number of genes. However, for a fixed number of individuals, the total number of features under study also has an impact on optimism. Results are shown in an example with *p *= 100 patients. Figure 5 shows the values of the differences between $\rho_{Tr}^{2}$ and ${\bar{\rho }}_{Te}^{2}$ in the transcriptomic model. When the total number of genes increased, Δ_*TrTe *_increased too. When there were too few genes of interest, it was difficult for the selection method to find the relevant ones (true positives). Genes selected on the training set had no predictive power on the test sets, which can be explained as follows. The more genes there are, the higher the optimism: the greater the number of genes under study, the more overestimated is the predictive power of the transcriptomic model. The high value of $\rho_{Tr}^{2}$ is due to noise and not to real information. The study of Δ_*TePop*_, indicated that genes selected on the training set are not able to relay the predictive power really contained in the test set when the number of genes truly related to survival is too small relatively to the total number of genes. Indeed, differences were negative, and increased with increasing *p*_1_.

## Discussion

Genes are not yet validated as predictors of outcome. They are selected on a single dataset and assumed to have the same predictive power with other datasets. But results show that this predictive power is overestimated in the case of genes. This overestimation is even more significant when the number of patients is low and the total number of genes high. This is due to two phenomena which overlap: the gene selection mechanism and the power problem. When there are too few patients the genes that are selected are not the true ones due to a lack of power. This problem is not encountered for clinical variables, for which the selection process is over because they have been already validated. The same problem arises when there are too many genes relatively to the number of genes truly related to survival. This problem is difficult to solve in real life studies and must be kept in mind. Indeed, the number of genes of interest is not known in advance.

Many papers dealt with the choice of the best method for gene selection. Our aim was not to study a specific method but rather to study what happens once genes have been selected. However, some comments may be made as to the TGD. First, from one dataset to another, there is considerable variety in the number and identity of genes in a selected set. Nevertheless, the number of true positives, which increases with the number of samples, is more stable. Note that the number of selected genes depends on the choice of the parameter *τ*. With a fixed value of *τ *and *p*_1_, the conclusions would be the same whatever the choice of this parameter: optimism increases with the number of patients and decreases with the number of genes involved in the study. Second, one point that appears to us as a drawback of this method is that it gives very low coefficient estimations, even for true positives. In our case, we only used the TGD for selection purposes, and coefficients of selected genes were re-estimated in a new Cox model. However, we noticed that some of these genes had very low estimated coefficients on the training sets, even more so with a low number of samples. We may wonder why these genes were selected.

To answer the question of comparative optimism of clinical and transcriptomic variables, we worked on simulated datasets reflecting the real situation encountered by clinicians and statisticians. Thus, we simulated only two true clinical variables, but many superfluous genes. Our conclusions depend on this choice of the simulation setup. It is not the nature (classical clinical or transcriptomic) of the two types of variables that explains the difference in the introduced optimism but their status (selected and validated or not): few validated variables for clinical variables and many variables under selection for genes, of which noisy variables. It is clear that with 50 clinical variables and only 5 biologically pre-selected relevant cancer genes, the situation would be reversed.

Concerning the simulation process, as the real correlation structure of genes is not well known, we chose not to include it in this work. Moreover, clinical variables and gene-expressions were all modeled to be independent. Future studies will aim to model dependence structures between the two types of variables and genes.

## Conclusion

By comparing the predictive power and optimism from clinical variables with genes two phenomena have to be taken into account: overestimation for genes due to the selection process and underestimation for clinical variables due to the omission of relevant genes. By including clinical and transcriptomic variables in the same model these results must be kept in mind. The predictive power of the clinical variables must not be neglected. In comparison with genes, their importance is not overestimated, which gives the feeling that they have less influence. In reality, part of their impact is hidden by the optimism encountered for genes.

## Authors' contributions

CT wrote the computer code for simulations, carried out the analysis, analyzed the results and drafted the manuscript. DM-B contributed to the design of the study, interpretation of the results, and contributed with PR to writing the manuscript. All authors read and approved the final manuscript.
